# Thermal transport across grain boundaries in polycrystalline silicene: A multiscale modeling

**DOI:** 10.1038/s41598-019-42187-w

**Published:** 2019-04-05

**Authors:** Maryam Khalkhali, Ali Rajabpour, Farhad Khoeini

**Affiliations:** 1 Department of Physics, University of Zanjan, Zanjan, 45195-313 Iran; 2 Mechanical Engineering Department, Imam Khomeini International University, Qazvin, 34148–96818 Iran

## Abstract

During the fabrication process of large scale silicene, through common chemical vapor deposition (CVD) technique, polycrystalline films are quite likely to be produced, and the existence of Kapitza thermal resistance along grain boundaries could result in substantial changes of their thermal properties. In the present study, the thermal transport along polycrystalline silicene was evaluated by performing a multiscale method. Non-equilibrium molecular dynamics simulations (NEMD) was carried out to assess the interfacial thermal resistance of various constructed grain boundaries in silicene. The effects of tensile strain and the mean temperature on the interfacial thermal resistance were also examined. In the following stage, the effective thermal conductivity of polycrystalline silicene was investigated considering the effects of grain size and tensile strain. Our results indicate that the average values of Kapitza conductance at grain boundaries at room temperature were estimated to be nearly 2.56 × 10^9^ W/m^2^ K and 2.46 × 10^9^ W/m^2^ K through utilizing Tersoff and Stillinger-Weber interatomic potentials respectively. Also, in spite of the mean temperature, whose increment does not change Kapitza resistance, the interfacial thermal resistance could be controlled by applying strain. Furthermore, it was found that by tuning the grain size of polycrystalline silicene, its thermal conductivity could be modulated up to one order of magnitude.

## Introduction

Nowadays, novel two-dimensional materials (2D Materials) have attracted widespread research interest due to their promising application potential in nanotechnology^1–4^. The successful exfoliation of graphene^5,6^, as a one-atom-thick planar sheet with fascinating properties^7–9^, has served as a milestone in experimental attempts to synthesize the new class of 2D materials^10–13^.

Silicene, a honeycomb structure of silicon elements, has emerged as a favorable monolayer material because of its outstanding physical and chemical properties^3,14,15^ as well as its compatibility with current silicon-based electronics^16^. Although theoretical possibility of silicene was predicted by Takeda *et al*.^17^ in 1994, it was successfully grown on various metal substrates in the recent decade^3,18–20^.

Owing to the structural similarity of silicene and graphene, silicene can be a satisfactory alternative to graphene. Furthermore, the slightly buckled structure of silicene and the existence of mixed sp^2^-sp^3^ hybridization lead to some new features^21^. For instance, despite graphene, the band gaps of silicene can be opened and tuned when exposed to an external electric field, proposing it as desirable building-blocks such as field effect transistors^22,23^, solar cells^24,25^, reusable molecule sensors^26^ and Li-ion batteries^27^. In addition, the physics in quantum phase transition can be explored with the interaction between the electromagnetic field and spin–orbit coupling in silicene^28,29^. Moreover, this slightly buckled structure will significantly alter the thermal conductivity of silicene due to breaking the symmetry of the out-of-plane direction. Thus, unlike graphene, silicene exhibits a low thermal conductivity^30^. Because the effective procedure to improve the thermoelectric performance is to reduce the thermal conductivity with maintaining the electronic transport features, silicene may show a great advantage as a promising thermoelectric material^30,31^.

It is notable that the reported distinguished properties of silicene belong to single crystal and defect-free samples, while various types of defects are typically formed during the fabrication process which may considerably affect electrical, mechanical, thermal, and other properties of silicene^32–34^.

Among all the developed fabrication methods, the chemical vapor deposition (CVD) is a common approach to synthesize different types of two-dimensional atomic crystals due to its simplicity, the potential large-scale application, high-quality production and its rather low expenses^35,36^. It is worth mentioning that CVD process inevitably lead to the formation of polycrystalline structures. In the polycrystalline morphology, grain boundaries appear where grains with different crystalline orientations face each other. The grain boundaries expand across the structure and because of their more distinct lattices than that of pristine grains, are regarded as topological defects. These topological defects can scatter the phonons and can also drastically change the thermal, mechanical and electronic features of the constructed polycrystalline product^37–39^. As a consequence, a fundamental understanding of the effects of grain boundary on the physical properties of polycrystalline samples is of crucial importance for the potential applications of these polycrystalline structures.

During the past decades, thermal transport exploration on 2D polycrystalline structure were often concentrated on graphene, hexagonal boron nitride and polycrystalline MoS_2_^40–42^. As an instance, Mortazavi *et al*.^43^ investigated the thermal transport of polycrystalline MoS_2_ by developing a multi-scale method. They first performed the molecular dynamics simulations in order to explore Kapitza thermal conductance of different types of grain boundaries, detectable in CVD constructed MoS_2_. Next, in order to study the effective thermal conductivity of polycrystalline samples at macroscopic level, they designed continuum models of MoS_2_ films utilizing the finite element method. Consequently, they found that thermal conductivity of samples can be modulated by changing their grain size. Bazrafshan *et al*.^44^ developed a combined NEMD atomistic-continuum multi-scale modeling to thermal transport engineering in polycrystalline graphene. They also examined the impact of nitrogen and boron doping, grain size, and mechanical strain on the effective thermal conductivity of polycrystalline graphene films. Their results indicated that Kapitza conductance and the thermal conductivity of polycrystalline graphene with nano-sized grains was not affected by nitrogen and boron doping. Also, they represented that the interfacial thermal resistance had a significant role in thermal transport within polycrystalline graphene with small grain sizes.

Although many potential applications are foreseen for silicene in the realm of thermoelectricity, thermal management and energy storage, to date, some limited number of research lines have been devoted to the thermal properties of polycrystalline silicene. As the pioneers of the field, Ju *et al*.^45^ performed the molecular dynamics simulations in order to explore the out-of-plane thermal conductivity of polycrystalline silicon nanofilm. They examined the effect of temperature, film thickness, and average grain size in the out-of-plane thermal conductivity of polycrystalline sample by the Muller-Plathe method. They observed that the polycrystalline thermal conductivity was lower than that of the silicon single crystal nanofilm and the out-of-plane thermal conductivity of polycrystalline silicon nanofilm was sensitive to the grain size.

Recently, Gao *et al*.^35^ carried out Green–Kubo equilibrium molecular dynamics simulations to measure the thermal conductivity of polycrystalline silicene with grain size of up to 50 nm. According to their report, polycrystalline silicene demonstrates extremely low thermal conductivity compared with both amorphous silicene and one-dimensional polycrystalline silicon nanowires with the identical grain size. They carried out phonon spectral energy density analysis and revealed that ultralow thermal conductivity of polycrystalline silicene originates from the phonon scattering through the boundary of grains.

As another instance, Roy *et al*.^46^ used the non-equilibrium molecular dynamics simulation to study the role of grain size in the thermal conductivity of polycrystalline Silicene. Their findings revealed that the thermal conductivity of the polycrystalline silicene with ultra-fine nano-grained was more sensitive to the grain size compared with a larger grain size sample. Therefore, with the increase in the grain size, the thermal conductivity increased and finally converged to a certain value.

Among few issues in polycrystalline silicene, which are based on molecular dynamics (MD) simulations, various investigations are concentrated on grains with small sizes. However, 2D polycrystalline structure, produced by CVD method, consists of grains with larger size (typically few hundred nanometers). Since the high computational costs of MD simulations limits the modeling of polycrystalline silicene with microscale grain sizes, we, in the current study, investigated the thermal properties of polycrystalline silicene through a multiscale modelling, consisting of non-equilibrium molecular dynamics simulations (NEMD) and continuum heat conduction method. We first applied classical NEMD simulations in order to compute the interfacial thermal resistance (Kapitza thermal resistance) of six different constructed grain boundaries in silicene. Following that, we intensively explored the effect of tensile strain and the mean temperature on the interfacial thermal resistance. Finally, based on the MD provided data, we made a continuum model of polycrystalline silicene and examine the effective thermal conductivity of the model by taking into consideration the effects of grain size and mechanical strain.

## Simulation Method

**Figure 1:**
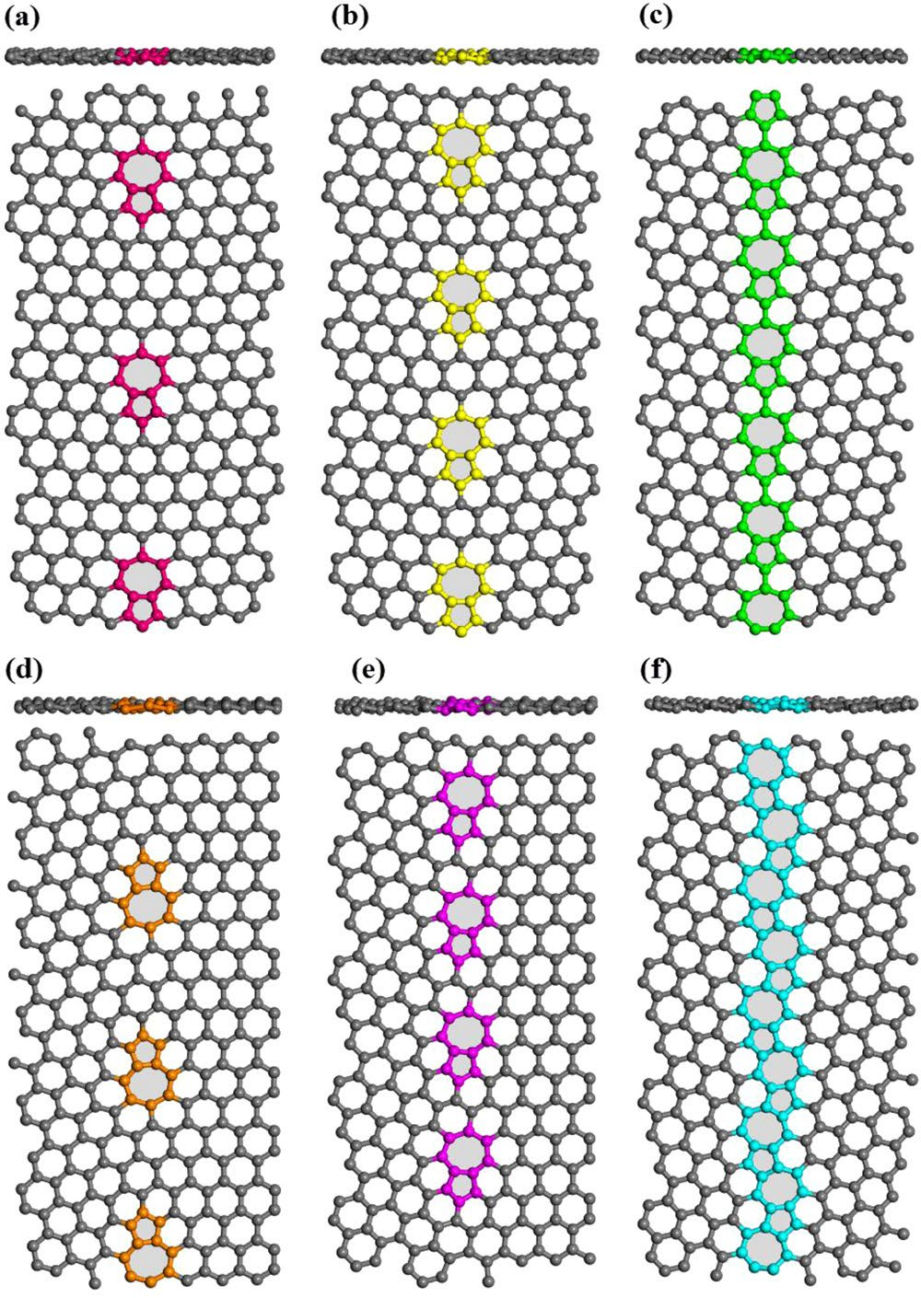
Top and side views of atomic structures of six different grain boundaries consisting of pentagon-heptagon defect pairs with different defect concentrations: (**a**) 5-7-6-6-s, (**b**) 5-7-6-s, (**c**) 5-7-5-7-s, (**d**) 5-7-6-6-a, (**e**) 5-7-6-a, and (**f**) 5-7-5-7-a.

Based on first-principles calculations^34,47–49^ and experimental evidence via high-resolution electron microscopy^50,51^, various types of dislocation cores may exist in the grain boundaries of the two-dimensional polycrystalline materials. It is worth noting that the existence of a wide variety of dislocation cores depends on grains orientation as well as distances between the atoms in the two sides of the grain boundary. Among these diverse configurations, we explored a common pentagon-heptagon defect pairs with different defect concentrations along the grain boundaries. Figure 1 illustrates the atomic structures of six different grain boundaries consisting of pentagon-heptagon defect pairs. Also, we evaluated both the symmetrical and asymmetrical grain boundaries for pentagon-heptagon defect pairs. As depicted in Fig. 1, for both the symmetrical and the asymmetrical grain boundaries, the defect concentration along the grain boundaries gradually increased. There were two hexagonal rings which separated the two 5–7 defect pairs for the least defective ones, and for the most defective cases, there existed no hexagonal rings which separated the two 5–7 dislocation cores. Also, all constructed structures were periodic along the grain boundary direction.

In this research, molecular dynamics simulation was performed using the Large Scale Atomic/Molecular Massively Parallel Simulator (LAMMPS) package^52^ in order to calculate the interfacial thermal resistance of various grain boundaries in silicene. In the MD method, the employment of appropriate potential functions is required for the accuracy of predictions. Previous studies have shown that Stillinger-Weber and Tersoff potentials have been widely utilized to evaluate the thermal properties of the silicon-based materials. Unfortunately, in the case of silicene, the commonly used Stillinger-Weber potential cannot maintain the structure of the silicene. Therefore, it is not proper for investigating the thermal conductivity of monolayer silicene. As a result, we employed optimized Stillinger-Weber potential which is recently reparametrized by Zhang *et al*.^31^ which can precisely reproduce the buckled structure of silicene and the phonon dispersion computed from ab initio. On the other hand, to evaluate the sensitivity of the results to the chosen interaction potential, we employed Tersoff^53^ potential as another potential to compare the results. In addition, Newton’s equations of motion were integrated via the velocity Verlet algorithm^54^ with a time step of 0.5 fs. In the next stage, the periodic boundary condition was employed in the X direction and the free boundary conditions were set in Y and Z directions.

In order to rearrange the atoms at grain boundaries and get the stable structures, energy minimization was performed and the systems were annealed from 1 to 10 K for 150 ps in an NVE ensemble using Langevin thermostat. Then, the structures were relaxed at room temperature (300 K) for 1 ns under NVT ensemble and coupling to Nose-Hover temperature thermostat. In order to generate the temperature gradient, and consequently, non-equilibrium heat current across the system, the sample was partitioned into 35 slabs along Y direction and the atoms at the two ends were fixed. Adjacent to these fix regions, there existed hot and cold slabs which were coupled to Nose-Hoover thermostat using NVT ensemble to set the temperature at T + ∆T/2 (315 K) and T − ∆T/2 (285 K) respectively. Besides, NVE ensemble was applied to the rest of the slabs. The mentioned condition was applied to the system so that the steady-state regime was achieved and led to a constant heat-flux. It is worth mentioning that the system was simulated for the entire10 ns and the first 4 ns were discarded as pre-equilibration step.

**Figure 2:**
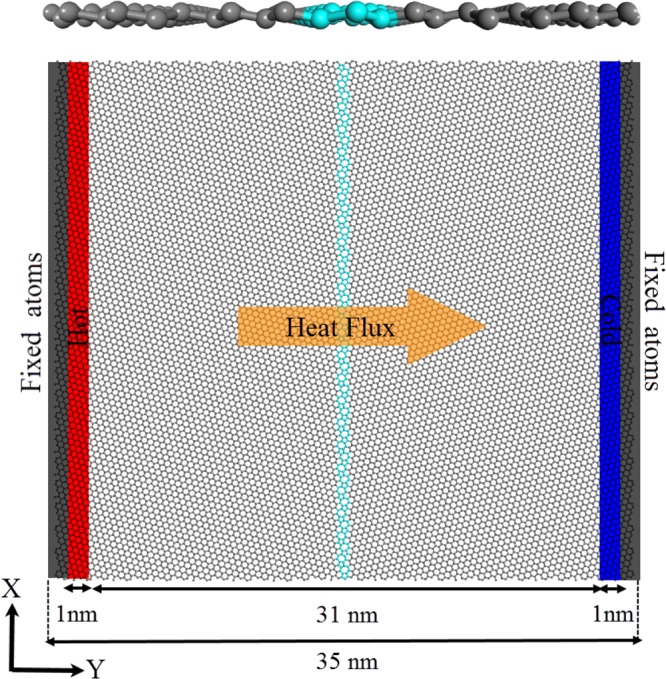
Molecular dynamics setup for calculation of the boundary resistance of the grain boundary. Top panel: the side view of the system with 5-7-5-7-a defect type. Bottom panel: the top view of the system with 5-7-5-7-a defects type.

Molecular dynamics setup for calculating the boundary resistance of the grain boundary is shown in Fig. 2. Considerable phonon scatterings at the grain boundary lead to a local temperature drop along the heat flux direction. The temperature drop at the grain boundary, Δ*T*_*GB*_, can be related to the heat flux according to the following equation^55,56^:

1 $${R}_{K}=\frac{{\rm{\Delta }}{T}_{GB}}{{J}_{Y}},$$

where *R*_*K*_ is known as interface thermal resistance or Kapitza resistance (discovered by P. L. Kapitza as a resistance to the heat flow across the interface between liquid helium and a solid)^57^. Δ*T*_*GB*_ can be obtained from the discontinuity of the 1D temperature profile along the sample length, and in order to calculate the heat flux along Y direction, *J*_*Y*_, the accumulative energy added and subtracted from baths were recorded every 1000 timesteps and plotted versus time. The slopes of the linear fitting to energy diagrams are equal to the heat current. We assumed the thickness of 4.65 Å for single-layer silicene.

Finally, the acquired interfacial thermal resistance values were used in two models: 1D thermal resistance model and continuum modeling for the exploration of effective thermal conductivity of polycrystalline silicene sheet.

In the 1D thermal resistance model, the thermal resistance of polycrystalline silicene sheet can be considered as the sum of the thermal resistance of the grain and the interfacial thermal resistance along the grain boundary according to the following equation^44^:

2 $$\frac{n\times GS}{{\kappa }_{eff}}=n\frac{GS}{{\kappa }_{G}}+(n-1)\frac{1}{{\kappa }_{GB}},$$

where n is the average number of grains along the Y direction, *κ*_*G*_ is the thermal conductivity of the grain, *κ*_*GB*_ is the Kapitza thermal conductance and GS is grain size which is defined as $GS=\sqrt{A/N}$ (A is the total area of the silicene sheet and N is the number of grains in polycrystalline silicene sample).

When the number of grains is very large (*n* → ∞), Eq. 2 can be simplified and effective thermal conductivity of polycrystalline silicene sheet can be calculated by^43^:

3 $${\kappa }_{eff}=\frac{{\kappa }_{G}\times {\kappa }_{GB}\times GS}{{\kappa }_{G}+{\kappa }_{GB}\times GS},$$

where an equal thermal conductivity of 41 W/mK (one of pristine silicene sheets)^58^ was assigned for all grains and the average value of the Kapitza thermal conductance for the six constructed grain boundaries -which are computed through the MD- is considered as the thermal conductance of the grain boundaries.

In the 2D heat conduction continuum model, we utilized Voronoi algorithm to produce polycrystalline silicene sheets with the grain size of 2 to 1000 nm. In this algorithm, some seeds (the number of seeds equals the number of grains) were randomly placed in the plane and atomic configuration with a random orientation was formed around each seed. Then, the distance between the generated atom and a seed in this grain was compared with other seeds. If this distance was the shortest one, this atom belonged to the grain, otherwise the generated atom had to be deleted^35^. After constructing the polycrystalline samples, finite element simulation was carried out.

At steady state, the heat equation was reduced to Fourier Law of Heat Conduction, therefore, in order to calculate the effective thermal conductivity, one dimensional form of the Fourier law $({\kappa }_{eff}={J}_{Y}\frac{L}{{\rm{\Delta }}T})$ was used, where *κ*_*eff*_ is the effective thermal conductivity of the sample and *J*_*Y*_ is the heat flux along Y direction.

As was already discussed, the thermal conductivity of 41 W/mK (the one of pristine silicene sheets)^58^ was assumed for all grains and in order to define the thermal conductance of the grain boundary, the NEMD results for six different grain boundaries averaged. Also in accordance with NEMD setup, temperatures at the left and right boundaries of polycrystalline silicene were set to *T*_*H*_ = 315 *K* and *T*_*C*_ = 285 *K* respectively. Besides, a triangular mesh was generated for the system and by numerically solving heat conduction via finite volume method, effective thermal conductivity of polycrystalline silicene sheet was obtained.

## Results and Discussions

In this study, we used a multiscale method consisting of non-equilibrium molecular dynamics simulations and solving continuum heat conduction equation in order to intensively investigate the effects of grain size and tensile strain on the thermal transport along the polycrystalline silicene.

**Figure 3:**
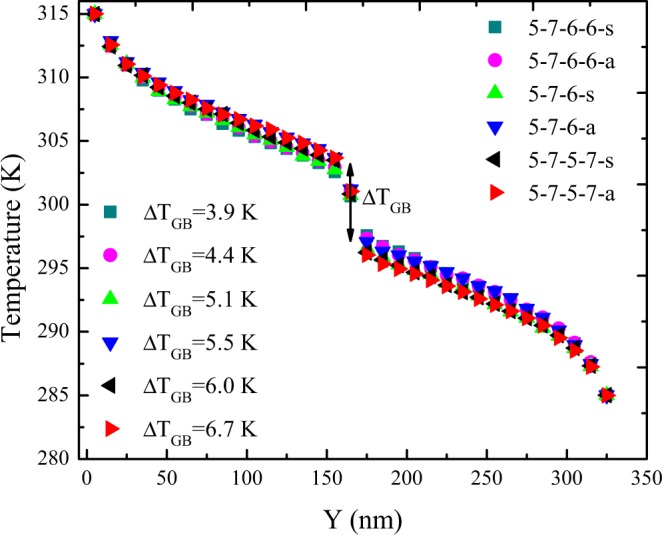
The steady-state 1D temperature profiles of different constructed grain boundaries along Y direction with the same lengths of 35 nm at T = 300 K and ΔT = 30 K.

The steady-state 1D temperature profiles of different constructed grain boundaries along with Y direction were presented in Fig. 3. A linear temperature gradient was observed in the area away from the heat baths. Additionally, in view of phonon scattering with the heat baths, there existed nonlinearity close the two ends. Also, the most distinguished features of the all temperature profiles were the discontinuity at the middle of the sample due to the existence of the grain boundary. The established temperature jump for all the samples are represented in Fig. 3. It can be seen that for both the symmetric and non-symmetric grain boundaries, with the increase in the defect concentration along the grain boundary, the temperature gap slightly increased. Furthermore, the temperature jump of the non-symmetric grain boundaries was explored to be higher than that of symmetric ones at the identical defect concentration.

**Figure 4:**
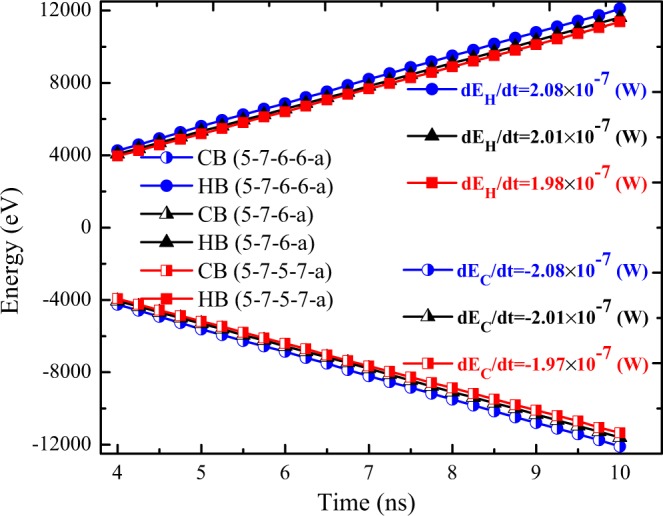
Accumulative added energy to the hot layer and subtracted energy from cold layer as a function of simulation time of the silicene films consisting of non-symmetric grain boundaries with different defect concentration at T = 300 K and ΔT = 30 K.

In Fig. 4, accumulative added energy to the hot segment and subtracted energy from the cold layer of the samples consisting of non-symmetric grain boundaries with different defect concentration are illustrated. Besides, the heat current $(\frac{dE}{dt})$ was computed for each sample as the slope of the linear fitted to energy profile. The results are depicted in Fig. 4. As expected, by increasing the defect concentration, the magnitude of energy flux slightly decreases. As it is observed, the gradient of added energy with respect to time for each sample is equal to its rate of subtracted energy, which confirms the conservation of energy in the NEMD simulations.

**Figure 5:**
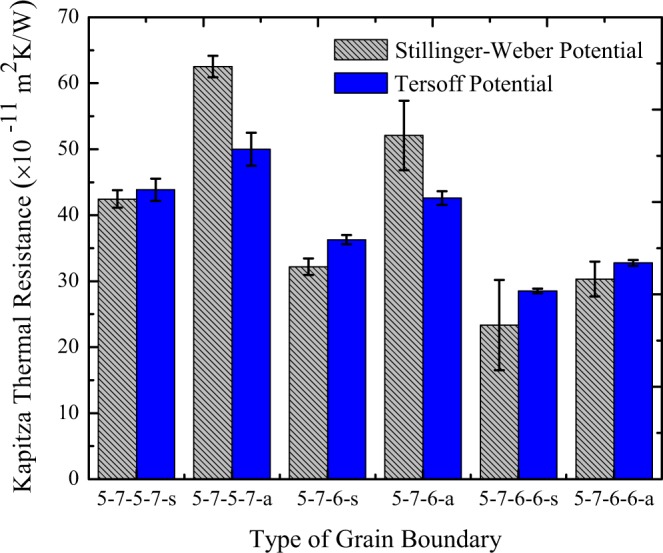
The interfacial thermal resistance of different constructed grain boundaries at T = 300 K and ΔT = 30 K using Stillinger-Weber and Tersoff MD potentials.

The interfacial thermal resistance of different constructed grain boundaries at room temperature is represented in Fig. 5. We used both Stillinger-Weber^31^ and Tersof^53^ potentials to explore the sensitivity of the results to the chosen interaction potential. The values of the Kapitza thermal resistance obtained from Tersoff potential for symmetric grain boundaries varies from 28.5 ± 0.3 (×10^−11^ m^2^ K/W) to 43.8 ± 1.6 (×10^−11^ m^2^ K/W) at the least and most defect concentration respectively. Furthermore, the amounts of Kapitza thermal resistance for non-symmetric grain boundaries ranges from 32.7 ± 0.4 (×10^−11^ m^2^ K/W) to 49.9 ± 2.4 (×10^−11^ m^2^ K/W) at the least and most defect concentration respectively. As expected, by increasing the defect concentration along the grain boundary the thermal resistance increases for both the symmetric and non-symmetric grain boundaries. The previous change happened because of the phonon-defect scattering. Also, it was found that the thermal resistance of each non-symmetric grain boundaries was higher than the symmetric one at identical defect concentration. This higher interfacial thermal resistance is because of the higher phonon–phonon scattering through the non-symmetric grain boundaries, which arises from the difference of the phonon spectrum in two sides of the grain boundary. The findings are both reliable and in line with the results of Mortazavi *et al*.^43^. Furthermore, Stillinger-Weber and Tersof potentials revealed the average values of Kapitza thermal conductance at grain boundaries at room temperature nearly 2.46 × 10^9^ W/m^2^ K and 2.56 × 10^9^ W/m^2^ K respectively.

In order to have a better understanding of the impact of grain boundary on the thermal transport of the sample, we calculated phonon density of states of two groups of atoms, belonging to two sides of the grain boundary. The phonon power spectral density was obtained by computing the Fourier transform of autocorrelation function of the velocity of atoms corresponding to two sides of the grain boundary as follow^59,60^:

4 $$P(\omega )=\sum _{i}\,\frac{{m}_{i}}{{k}_{B}{T}_{MD}}\,{\int }_{0}^{{\rm{\infty }}}\,{e}^{-j\omega t} < {v}_{i}(t).{v}_{i}(0) > dt,$$

where *ω* is the angular frequency, *m*_*i*_ is the mass of atom i and *v*_*i*_ is the velocity of the *i*th atom.

**Figure 6:**
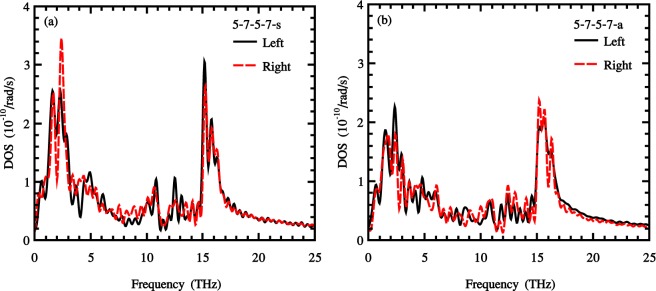
Phonon power spectral density of states at two sides of 5-7-5-7-s (**a**) and 5-7-5-7-a (**b**) grain boundaries at T = 300 K.

Figure 6(a,b) show the phonon density of states of two sides of the most defected symmetric and non-symmetric samples (5-7-5-7-s and 5-7-5-7-a). As illustrated in figures, there exist mismatches between the two spectra of left and right sides of the grain boundary for each samples. Thus, it can be concluded that the interfacial thermal resistance leads to the phonons scattering through the interface, which is in line with previous results in the literature^61^.

**Figure 7:**
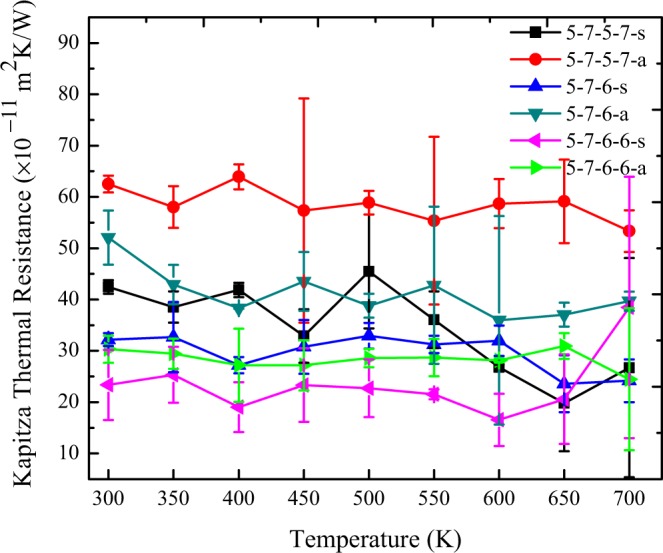
The Kapitza thermal resistance of different constructed grain boundaries as a function of mean temperature using the Stillinger-Weber potential; ΔT = 30 K.

In order to explore the sensitivity of the Kapitza thermal resistance to the mean temperature in silicene grain boundaries, we raised the mean temperature from 300 to 700 K using the Stillinger-Weber potential. In Fig. 7, the NEMD predictions for the interfacial thermal resistance of various constructed grain boundaries as a function of mean temperature are demonstrated. It is found that by increasing the temperature up to 700 K, no significant alteration was observed in the Kapitza thermal resistance of various silicene grain boundaries. The interfacial thermal resistance in all six types of grain boundaries depicted no changes, and a slight fluctuation was in the range of the statistical uncertainties of calculations. It is worth mentioning that Jhon *et al*.^62^ reported that the boundary resistance in polycrystalline graphene decreased as the temperature increased, but it is not found in the case of poly crystalline silicene. The reason could be the differences of silicene with graphene in terms of thermal properties. Also, as discussed before, the 5-7-5-7-a, which is the most defective non-symmetric sample, exhibits the highest interfacial thermal resistance and 5-7-6-6-s that is the least defective symmetric structure illustrates the lower Kapitza resistance.

Studying the interfacial thermal resistance of silicene grain boundaries under an extreme condition such as strain is required for empirical applications. Therefore, in this step, we investigated the effect of tensile strain on the Kapitza thermal resistance of the constructed grain boundaries.

The strain is defined as^63^:

5 $${{\rm{\varepsilon }}}_{y}=\frac{dL}{L},$$

where *L* is the initial length of the silicene sheet and *dL* is the change in length because of stretching one or both ends of the sample along the Y-direction. For imposing the tensile strain, the first segment of the silicene sheet was fixed and the last segment started to stretch along the sheet length with the stretching velocity of 0.005 Å/ps.

**Figure 8:**
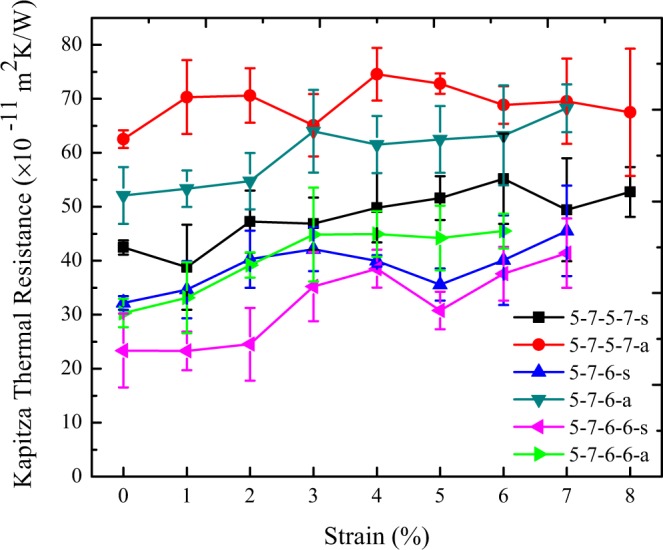
The interfacial thermal resistance of six constructed silicene grain boundaries under tensile strain at T = 300 K and ΔT = 30 K.

In Fig. 8, the results of NEMD simulations for constructed grain boundaries under the tensile strain are illustrated. It should be further said that, we also applied strain from 0.01 to 0.08 to each sample but critical value of tensile strain in some structures was less than 0.08. For instance, the 5-7-6-6-a structure stands tensile strain just up to 0.06. It was found that the tensile strains, which were applied to the heat flux directions, increases the Kapitza thermal resistance of the silicene grain boundaries and the minimum interfacial thermal resistance of the all samples is approximately found in strain-free constructions. Table 1 indicates the values of imposing tensile strain which causes the maximum increment in the Kapitza thermal resistance of the constructions.

**Table 1:** Values of imposing tensile strain which causes the maximum increment in the Kapitza thermal resistance of the samples.

Grain Type	5-7-5-7-s	5-7-5-7-a	5-7-6-s	5-7-6-a	5-7-6-6-s	5-7-6-6-a
Strain	0.06	0.04	0.07	0.07	0.07	0.06
Increment of the Kapitza resistance (%)	30	19	41	31	77	50

**Figure 9:**
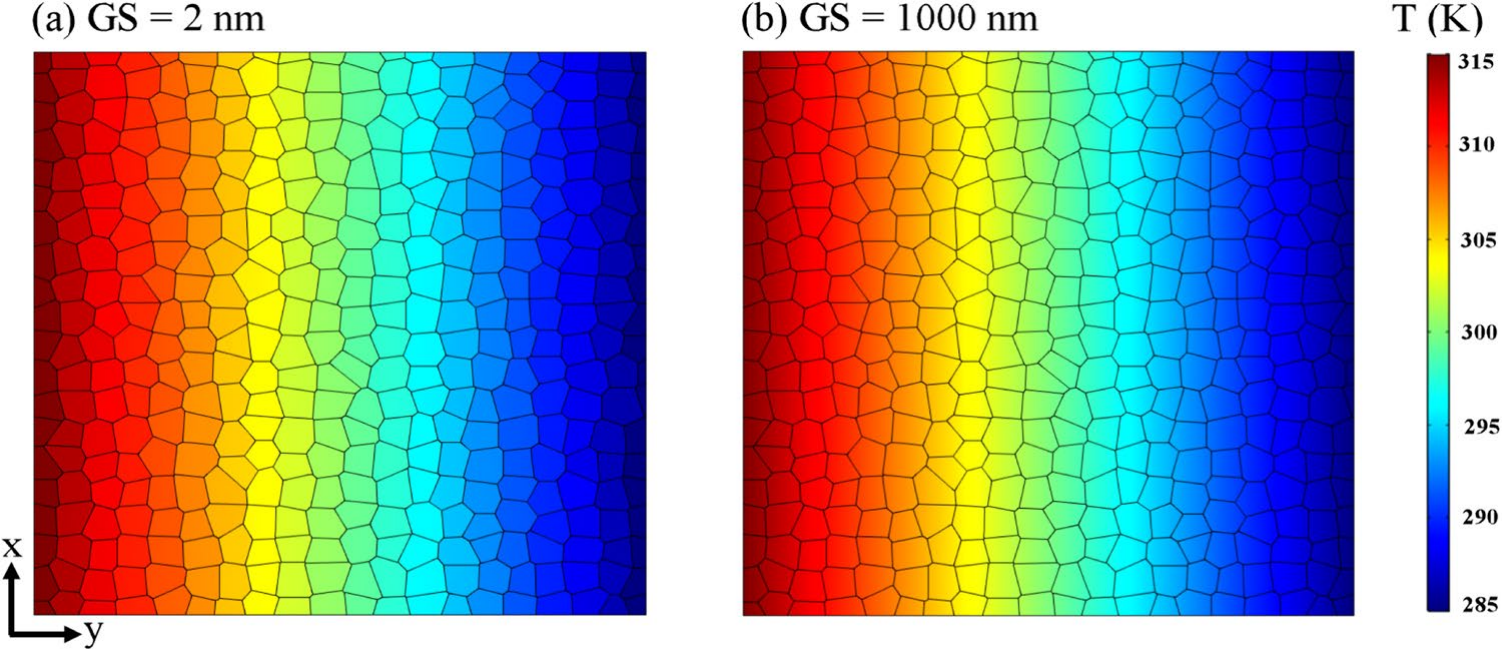
NEMD/2D continuum multiscale modeling results for the comparison of grain size effect on the temperature profile for polycrystalline silicene with grain sizes of 2 nm (**a**) and 1000 nm (**b**) at T = 300 K and ΔT = 30 K; *κ*_*G*_ and *κ*_*GB*_ were set to 41 W/mK and 2.46 × 10^9^ W/m^2^ K respectively.

Figure 9 represents the temperature profiles for two polycrystalline silicene sheet with grain sizes of GS = 2 and 1000 nm, considering a grain thermal conductivity of *κ*_*G*_ = 41 W/mK and a Kapitza thermal conductance at grain boundaries of 2.46 × 10^9^ W/m^2^ K. It is obvious that for a sample with small grain size of 2 nm, the temperature distribution is approximately uniform inside each grain and a constant temperature can be assigned to individual grains. In contrast, for polycrystalline silicene film with large grain sizes, the temperature profile is not uniform inside each grain and a temperature gradient can be almost seen inside individual grains. These observations imply the fact that at grain sizes lower than 10 nm the thermal resistance at grain boundaries dominates over the grains’ thermal resistance but when the grain size increases, the effect of grain boundaries resistance is considerably weakened.

**Figure 10:**
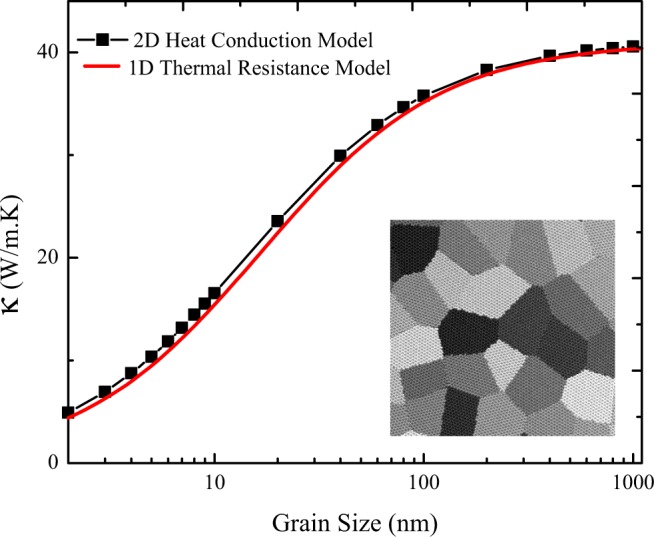
The effective thermal conductivity of polycrystalline silicene with various grain sizes based on 2D heat conduction and 1D thermal resistance models grain sizes are set to 1, 2, 3, …, 10, 20, 40, …, 100, 200, 400, …, 1000 nm.

In the present study, the thermal conductivity of polycrystalline silicene as a function of grain size was evaluated by the 2D continuum multiscale model and 1D thermal resistance approach. In Fig. 10, the effective thermal conductivity of polycrystalline silicene with various grain sizes based on proposed models are represented. It can be observed that the effective thermal conductivity of polycrystalline silicene exhibits increment with an increase in the sizes of grains. Furthermore, for samples with the grain sizes lower than 100 nm, the thermal conductivity increases drastically by increasing the grain size, but by increasing the grain size in the range of 100 to 1000 nm, the increase in the thermal conductivity declines and eventually achieves a plateau, close to the thermal conductivity of pristine silicene. As mentioned earlier, since we supposed identical thermal conductivity for all grains (41 W/mK)^58^, the key factor which assign the effective thermal conductivity of polycrystalline film is thermal conductance of the grain boundaries. Besides, it is demonstrated that when the grains sizes increase, the thermal conductivity of polycrystalline silicene changes nearly from 4.9 to 40.5 W/mK, specifying that by tuning the grain size of polycrystalline silicene, its thermal conductivity can be modulated up to one order of magnitude.

Also, as represented in Fig. 10, the results based on 1D thermal resistance approach are in line with findings of 2D continuum multiscale model showing more suitability of the 1D thermal resistance model due to its simplicity. Our results are in line with refs^43,44^.

**Figure 11:**
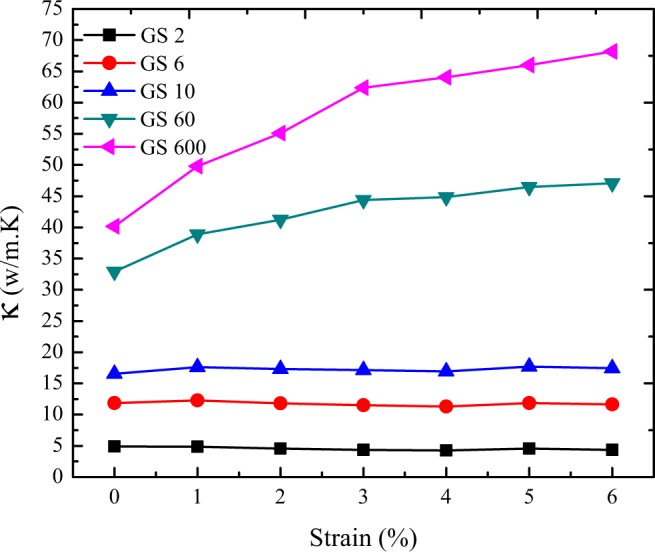
The thermal conductivity of polycrystalline silicene with different grain size of 2, 6, 10, 60 and 600 nm under tensile strain at T = 300 K and ΔT = 30 K.

Eventually, we examined the effect of tensile strain on the thermal conductivity of polycrystalline silicene. To this aim, we used a NEMD-2D continuum multiscale model. Figure 11 depicts the thermal conductivity of polycrystalline silicene with different grain size of 2, 6, 10, 60 and 600 nm under tensile strain from 1% to 6%. It is important to declare that for the grains, we assumed the thermal conductivity of under strain pristine silicene^58^ and for the thermal conductance of grain boundaries, we averaged the NEMD results for the under strain constructed samples. As it is represented, when the grain size increases, polycrystalline silicene exhibits thermal response to tensile strain similar to pristine silicene, in a way that the thermal conductivity of polycrystalline silicene with grain size of 60 and 600 nm, increase with applying tensile strain up to 6%. This distinct response of silicene to tensile strain can be attributed to its buckled structure. When silicene started to stretch, at first its bucked structure started to become less buckled and in-plane stiffness of silicene increased. Consequently, the thermal conductivity enhanced. Following that, with more increase in the tensile strains, the buckled structure of silicene would become flattened; thus, the in-plane stiffness of silicene decreased and led to the decrement of the thermal conductivity.

It is obvious that for the polycrystalline silicene with grain size smaller than 10 nm, by applying the strain up to 6%, no remarkable changes were observed on the effective thermal conductivity. As discussed earlier, the grain boundaries play the main role on the thermal conductivity of polycrystalline silicene with grain sizes smaller than 10 nm, and the dominant factor that defines the thermal conductivity is the thermal conductance of the grain boundaries^43,44^.

## Conclusions

In the present study, the thermal transport through polycrystalline silicene with performing a multiscale method consisting of classical NEMD and solving continuum heat conduction equation were intensively explored.

Extensive NEMD simulations were conducted so as to investigate the interfacial thermal resistance of six various constructed grain boundaries in polycrystalline silicene, as well as to examine the effects of tensile strain and the mean temperature on the thermal resistance. The average values of Kapitza conductance at grain boundaries were computed as almost 2.56 × 10^9^ W/m^2^ K and 2.46 × 10^9^ W/m^2^ K by using Tersoff and Stillinger-Weber interatomic potentials respectively. Furthermore, the results revealed that increasing the mean temperature of the sample does not affect the Kapitza resistance of the grain boundaries. Also, it was found that the interfacial thermal resistance of the grain boundaries can be tuned by applying tensile strain.

In order to examine the thermal properties of polycrystalline silicene film, the continuum model of polycrystalline silicene was constructed and its effective thermal conductivity was explored by taking the effects of grain size and tensile strain into consideration. It is worth mentioning that the NEMD obtained results were used to determine the thermal conductance of grain boundaries of polycrystalline sample. The results revealed that the thermal conductivity of polycrystalline silicene changes from 4.9 to 40.5 W/mK for the grains sizes of 2 to 1000 nm. Consequently, the effective thermal conductivity of polycrystalline silicene is adjustable by one order of magnitude with tuning its grain size. Also, the acquired results of the 2D continuum multiscale model were compared with those acquired from 1D thermal resistance approach, through which an acceptable agreement was observed.

Moreover, the thermal conductivity of polycrystalline silicene with large grain size (60 and 600 nm) increased after applying tensile strain up to 6%. For the sample with small grain size (less than 10 nm), the strain did not affect the thermal conductivity because the thermal conductance of grain boundaries plays a crucial role in the thermal transport properties of polycrystalline samples.
